# Supervised Machine-Learning Methodology for Industrial Robot Positional Health Using Artificial Neural Networks, Discrete Wavelet Transform, and Nonlinear Indicators

**DOI:** 10.3390/s23063213

**Published:** 2023-03-17

**Authors:** Ervin Galan-Uribe, Juan P. Amezquita-Sanchez, Luis Morales-Velazquez

**Affiliations:** Facultad de Ingeniería, Universidad Autónoma de Querétaro, Campus San Juan del Río, Río Moctezuma 249, Col. San Cayetano, San Juan del Río 76807, QRO, Mexico

**Keywords:** robot health, accuracy degradation, fault prognosis, principal component analysis, Katz fractal dimension, discrete wavelet transform

## Abstract

Robotic systems are a fundamental part of modern industrial development. In this regard, they are required for long periods, in repetitive processes that must comply with strict tolerance ranges. Hence, the positional accuracy of the robots is critical, since degradation of this can represent a considerable loss of resources. In recent years, prognosis and health management (PHM) methodologies, based on machine and deep learning, have been applied to robots, in order to diagnose and detect faults and identify the degradation of robot positional accuracy, using external measurement systems, such as lasers and cameras; however, their implementation is complex in industrial environments. In this respect, this paper proposes a method based on discrete wavelet transform, nonlinear indices, principal component analysis, and artificial neural networks, in order to detect a positional deviation in robot joints, by analyzing the currents of the actuators. The results show that the proposed methodology allows classification of the robot positional degradation with an accuracy of 100%, using its current signals. The early detection of robot positional degradation, allows the implementation of PHM strategies on time, and prevents losses in manufacturing processes.

## 1. Introduction

Robotic systems are an essential part of modern industrial revolution 4.0 manufacturing. Among the most common tasks for industrial robots, are pick-and-place, welding, machining parts, and painting [1]. A critical requirement, to perform these tasks with a high level of precision and quality, is the accuracy of the robot in positioning and orienting itself in the required positions, and repeating the operation continuously for long periods, with the least possible error. Ideally, once the robot has been programmed to perform a repetitive task, it should stay within its trajectory; however, in reality, various factors such as robot assembly failures, manufacturing defects in the structure, and backlash, can cause sudden or gradual degradation of the accuracy of the robot [2].

Prognostics and health management (PHM), is an engineering research area whose purpose is to reduce maintenance time to a minimum, through diagnostic monitoring and detection of anomalies and failures [3]. PHM is focused on analysis at the level of components present in a more complex system. In the case of a robotic system, the components to analyze would be the mechanical components present in the mechanical transmission stage and the electrical components. On the other hand, PHM centers on a system-level analysis, that considers factors such as the performance and operation of the system itself, and the environment in which it is located [4]. Different PHM strategies applied to robot manipulators have been developed at the component level. For example, in [5], a methodology was proposed to diagnose failures in the ballscrew of a robot, using time-frequency transform methods such as short-time Fourier transform (STFT), and wavelet packet transform (WPT), statistical indicators such as energy, root mean square (RMS), and kurtosis, and to perform a comparison between the classification methods: convolutional neural networks (CNN), logistic regression (LR), and k-nearest neighbors (KNN). Identifying the failure in a rotate vector reducer (RV), by analyzing acoustic emissions (AE), was presented in [6]. For this, they used WPT, to denoise the acoustic signals, and used a hidden Markov model (HMM), to infer the failure state of the RV. On the other hand, in [7], they analyzed a failure in the RV of a robot, using discrete wavelet transform (DWT), to calculate features such as wavelet energy, Shannon wavelet entropy, and statistical indicators. These are used by the algorithms, linear discriminant analysis (LDA), fine tree (FT), Naive Bayes (NB), and support vector machine (SVM), for fault classification. The simulation of the torque degradation of the actuator of an industrial robot, was presented in [8], and was carried out considering three faults: boundary degradation torque fault, boundary degradation torque rate fault, and proportional degradation of torque fault, concluding that the robot controller does not meet the control requirements to deal with a failure such as torque degradation. In [9], the classification of wave gear reducer faults and the eccentricity-bearing fault in an RV were carried out, each one by a different robot. A deep scattering spectrum (DSS) was used to extract scattering and scalogram coefficients, that are used as feature vectors for the NB, SVM, discriminant analysis (DA), KNN, ensemble learning, and decision tree (DT) classification algorithms. Other works related to PHM robotics, at the component level, can be consulted in gears [10,11,12,13], and drive belt looseness [14].

PHM strategies focused at the system level, have been presented, as in [15], where the normal condition, arm backslash, and overload faults were identified, in a hybrid robot using vibration signals. These were processed with different dimensional reduction methods, such as principal component analysis (PCA), locality preserving projection (LPP), and isometric feature mapping (ISOMAP). The obtained features in each reduction method are later united and reduced again using treelet transform (TT), then classified with NB. In [16], a methodology was proposed to detect anomalies in the overload in the robot end-effector, and its effect on the joints, using contrived failure data and the random forest regression (RFR), support vector regression (SVR), and deep neural network regression (DNNR) for the prediction of the condition in the robot. The theoretical pose coordinates and actual pose error, to train a deep belief network to model the positioning error of an industrial robot and improve its positional and orientation accuracy, were presented in [17]. A methodology for monitoring the health status of an industrial robot, using the standard deviation of the torque, power, and position deviation signals as indicators, was presented in [18].

Within the PHM strategies at the system level, research has been carried out focused on developing methodologies for the robot positional quick health assessment [19], which evaluated the accuracy of the position and orientation of the robot. In this sense, different works have been presented, e.g., [2] where an advanced error model was carried out, to characterize dependent errors present in the robot (deflections of the structure and non-ideal motions), using Chebyshev polynomials. The robot position was measured by means of an artificial vision system, designed for this purpose and presented in [20]. In [21], a methodology based on deep long short-term memory (DLSTM) and regression adjustment multivariate (RAM) was proposed, to model and predict (respectively) the displacement of an industrial robot, and estimate the residual error in the tool center position (TCP). A smart target was designed, to be used with the vision system mentioned before, and its feasibility in performing robot accuracy assessment was verified in [22,23]. In [24], a methodology was presented for integrating top-level and component-level PHM (external measurement system and controller, environmental, and add-on sensing, respectively), for accuracy monitoring and diagnosis of the robot tool center. The top-level PHM allows for identifying the deviation of the TCP. In contrast, the component-level PHM allows one to carry out an analysis to know the cause of the deviation. An optical tracking system to improve the accuracy of the robot was presented in [25], and a method for re-calibration of the robot through kinematic analysis was proposed.

It should be noted, that some of the proposals for robot positional health assessment use a system (cameras or lasers) external to the robotic system, to measure TCP. Although this system can be used in controlled conditions and works correctly, it is complex to implement in an industrial environment. Some factors that condition the use of external measurement systems in industrial facilities include vibrations, noise, reflective materials, lighting, and accessibility, in addition to the logistics of analyzing a large group of robots. In this sense, using PHM methodologies based on artificial intelligence and robot information obtained at the controller level, is proposed, to obtain an agile methodology for robot positional quick health assessment. The advantage of using information from the controller, is its rapid access and ability to join the information from several robots that perform the same operation, in a dataset that provides robustness to machine learning techniques. This reduces the diagnostic time, by not instrumenting each robot with an external monitoring system. Machine learning techniques can classify failures of different types and, once trained, can be used to analyze information from other robots that perform the same task.

Based on the previously mentioned literature, the contribution of this work is a methodological proposal, based on supervised machine learning for robot positional quick health assessment, using current signals from the joints of the robot to determine if the joint presents an anomalous behavior that favors the deviation in the end-effector. To do this, a dataset [26] consisting of the current signals from the motors when following a trajectory immediately after powering on (cold start), and after running for more than 2 h (hot operation), is used. Ideally, the current signals in both cases should be similar (without deviation). However, a change in the currents over time can cause the robot joint to drift. For this problem, the original signal, at different noise levels (5 dB, 10 dB, 15 dB, and 20 dB) is used, to give robustness to the method, and the time-frequency analysis of the current signals is carried out using DWT. Subsequently, the energy index and Katz fractality dimension are calculated for each decomposition level. Then, a reduction in the information, through principal component analysis, is made for each feature. Finally, a feed-forward neural network is trained, using each feature to classify each joint.

The results obtained with the proposed methodology, for all noise level signals and energy indexes, have an accuracy greater than 97%, for classifying current signals. However, results of 99% are obtained when the Katz fractality dimension is used to train the neural network. When individual noise level signals are used for classification, an accuracy of 100% is obtained for energy and Katz classifiers, and a simple expert system is proposed to achieve a reliable result. Since both classifiers have 100% accuracy, the expert system also has 100% accuracy. These results, and the low computational time needed for its implementation, show that the proposed method is valid for a quick health positional assessment for robots.

## 2. Materials and Methods

### 2.1. Discrete Wavelet Transform

The discrete wavelet transform (DWT) is a signal processing technique that provides signal time-frequency information [27]. In a simplified way, the signal is passed through a series of high-pass and low-pass filters, in which a separation is made into corresponding frequency bands. To carry out the operation, the convolution between the signal of interest and a transformation function, called “mother wavelet”, is carried out, which establishes the parameters of the filters.

Using the algorithm of Mallat, the DWT produces a new signal information representation, which can be extended to multiple decomposition levels for different frequencies.

The DWT is defined by Equation (1), where x(k) represents a discrete-time signal of *k* samples and ψ to the discrete mother wavelet, for the decomposition level *i*.

(1) $$DWT_{ik}=\sum x\left(k\right)\psi_{i,k}\left(t\right)$$

Different wavelet mothers exist, such as Haar, Daubechies, Meyer, and Mexican hat. Due to the different properties of the mother wavelets, the choice of which one to use depends on the specific application [28]; in this work, the discrete Meyer mother wavelet is used, due to its properties of, a fast decrease to zero, not producing aliasing errors, and being infinitely differentiable [29] orthogonally, thus conserving the energy of the signal and compact support in the frequency domain [30].

To calculate the DWT, the signal first passes through a low-pass filter, g[n], with unit impulse response, as shown in Equation (2), and is also passed through a high-pass filter, h[n], in the same way.

(2) $$y\left[n\right]=\left(x*g\right)\left[n\right]=\sum_{k=-\infty }^{\infty }x\left[k\right]g\left[n-k\right]$$

**Figure 1:**
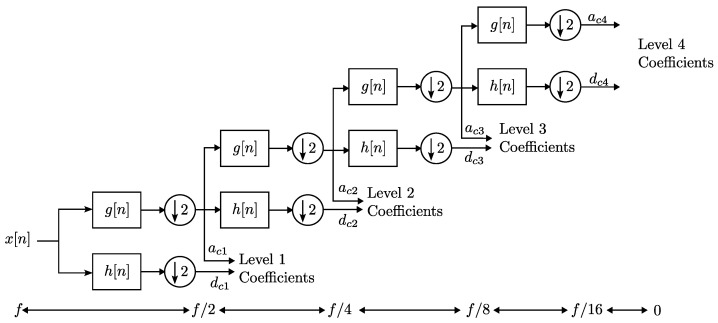
Representation of the Mallat algorithm for a decomposition level four. In the lower part, it is shown how the frequency of the signal is divided in each decomposition level.

By performing the above filtering, the signal frequency is separated into high and low-frequency bands, and the number of signal samples can be halved (sub-sampled by two). The result obtained by the low-pass filtering, g[n], is processed again, with the filters h[n] and g[n], and sub-sampling by two, obtaining two more frequency bands. This process can be repeated until the required decomposition level is achieved, shown in Figure 1. The decomposition level is limited by the number of signal samples and sample frequency, $f_{s}$. The bandwidths of each *i* level can be calculated with the following Equations (3) and (4):

(3) $$a_{ci}=\left[0,\frac{fs}{2^{i+1}}\right],$$

(4) $$d_{ci}=\left[\frac{fs}{2^{i+1}},\frac{fs}{2^{1}}\right].$$

Some recent applications of the DWT are, fault detection in electrical machines [31,32] and analysis of electroencephalographic signals (EEG) from patients with diseases such as Parkinson’s [33].

### 2.2. Energy Index

The amount of energy, *E*, that a signal contains in it, is defined by Equation (5), as the area under the squared magnitude of the signal [34]

(5) $$E=\sum_{k=1}^{n}{\left|x\left(k\right)\right|}^{2},$$

where *n* is the number of samples of the signal.

In this work, for the normalization of the index, it is proposed to calculate the energy of each approximation and detail obtained by applying the DWT to the original signal, as shown below in Equations (6) and (7).

(6) $$Ea_{i}=\sum_{k=1}^{n}{\left|a_{i}\left(k\right)\right|}^{2},$$

(7) $$Ed_{i}=\sum_{k=1}^{n}{\left|d_{i}\left(k\right)\right|}^{2}.$$

From the previous equations, *a* and *d* represent the decomposition of approximation and detail, respectively, and *i* is the level of decomposition.

The normalization of the energy indices proceeds as the ratio between the total energy of the signal and the energy of the level of decomposition of approximations and details, as shown below in Equation (8) and (9):

(8) $$E_{ai}=\frac{Ea_{i}}{E},$$

(9) $$E_{di}=\frac{Ed_{i}}{E}.$$

The change in the magnitude of the energy index over time would be an indicator of a possible joint deviation of the robot. Furthermore, the energy index calculated from the DWT coefficients is recommended in works such as [35] (on EEG signals), for finding useful information that cannot be observed in the raw signal, as well as its low computational complexity.

### 2.3. Katz Fractal Dimension

This method was presented by Katz, in 1988 [36]. It is proposed that the fractality dimension of a waveform (planar curve) of size *N*, can be estimated by sampling its points and the distances between them.

The Katz fractal dimension of a signal, with size *N*, is defined in Equation (10) as:

(10) $$D_{K}=\frac{\log\left(n\right)}{\log\left(\frac{d}{L}\right)+\log\left(n\right)},$$

where n=N−1, while the *d* term, which represents the planar extension of the curve, is defined as d=max[dist(1,i)]. Then the operator dist[] represents the distance between the initial point and the others (for curves that do not cross themselves). Finally, the term *L*, which represents the length of the curve, is defined in Equation (11) as:

(11) $$L=\sum_{i=1}^{n}dis\left[i,i+1\right].$$

For a time series, the fractal dimension provides information about self-similarity, that is, how much a series is composed of smaller versions of itself [37]. When analyzing current signals with fractal dimension, changes in the signal waveform over time can indicate a deviation in joint position, and cause positional degradation of the robot’s TCP. For this reason, and its easy implementation [38], the use of the Katz fractal dimension is proposed in this work. This index has been used in different applications, such as the detection of faults on induction motors [38], the study of the geoelectric field of the earth during severe geomagnetic storms [39], and speech emotion classification [40].

### 2.4. Principal Component Analysis

Principal component analysis (PCA) is a technique used to reduce the dimensions of a dataset. Pearson proposed it in 1901 [41] and it has been widely used ever since.

The original data is deconstructed into eigenvalues (magnitude) and eigenvectors (direction), on which a projection is made, to obtain a new representation of the information. The projection can be made in different numbers of axes, which correspond to the number of required dimensions, and are called principal components.

In these, the representability of the original data is distributed in descending order, so the first principal components concentrate the most significant amount of information from the original data. This reduction in information facilitates the manipulation and visualization of the data, reducing the space occupied for them in memory.

The PCA algorithm can be summarized as follows:

- Suppose a dataset with column vectors of the form $X_{n}=\left[x_{1},x_{2},\cdots ,x_{n}\right]$.
- Calculate the average ${\bar{x}}_{i}$ for each of the vectors $x_{i}$ and subtract them for each element of the same ${\hat{x}}_{i}=x_{i}-{\bar{x}}_{i}$. Define ${\hat{X}}_{n}=\left[{\hat{x}}_{1},{\hat{x}}_{2},\cdots ,{\hat{x}}_{n}\right]$.
- Generate the covariance matrix $B={\hat{X}}^{T}\hat{X}$.
- Find the eigenvectors and eigenvalues of *B*. Sort the eigenvalues in descending order, and apply the same order to the eigenvectors.
- To reduce the original data to a smaller dimension *k*, select *k* eigenvectors and construct a matrix $V_{k}$.
- Finally, the new representation of data *Z*, in *k*-dimensions, is obtained by means of $Z=V_{k}^{T}B$.

For a complete review of the algorithm, it is recommended to consult the following references [41,42].

### 2.5. Artificial Neural Networks

**Figure 2:**
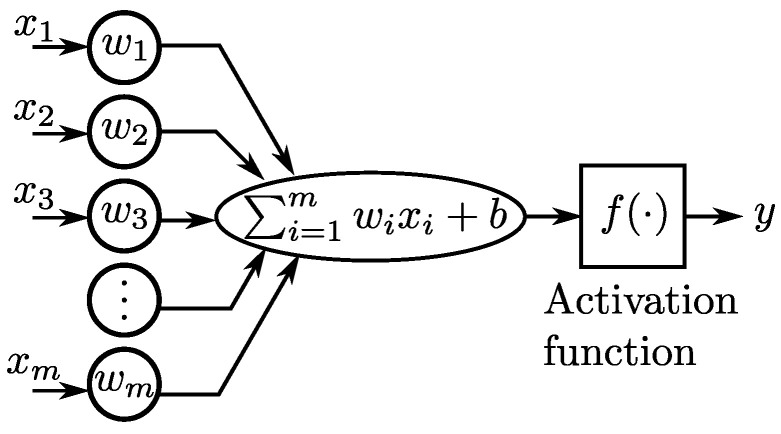
Diagram of a simple perceptron.

Artificial neural networks (ANN) are a mathematical imitation of the process carried out by neurons in the human brain. The primary element of an ANN is the simple perceptron, shown in Figure 2. This mathematical element is the equivalent of a neuron, where a vector input *x*, of size *m*, is multiplied by a weight vector *w*, then all the products are added to a bias coefficient *b*, and the result of this operation is passed to the activation function f(·) (e.g., binary step, linear, sigmoid, and hyperbolic tangent) to provide the output *y*. A set of interconnected simple perceptrons gives rise to an ANN.

The different ANN architectures are given by how the neurons connect in the different stages. The feed-forward and feed-backward neural networks (FFNN and FBNN, respectively) are the two main divisions from which the other variants arise, such as multi-layer perceptron (MLP) and Bayesian-regularized neural networks (BRANN), among others. Particularly, MLP has the characteristics of ease of implementation, low training time, and providing high-quality models [43]. Due to this, its use is proposed in this work.

**Figure 3:**
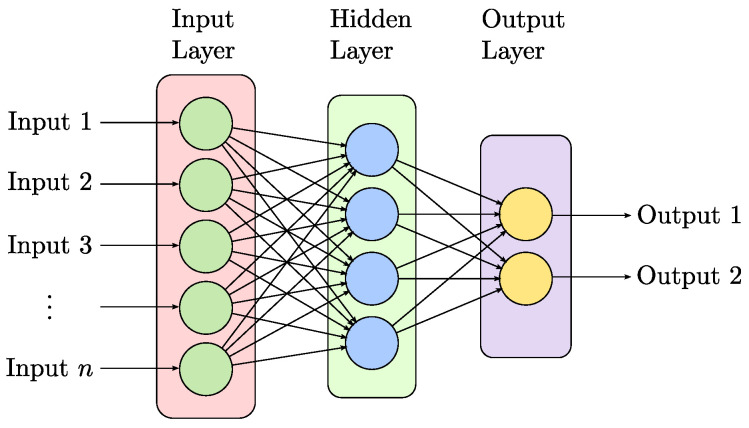
General diagram of an artificial neural network, the connections between layers display a feed-forward neural network (FFNN).

A simple ANN comprises three sections, the input stage, the hidden layer, and the output stage (Figure 3). In the input stage, the information to be analyzed is delivered to the neural network. In the hidden layer, that information is processed, and the result is delivered in the output. ANNs are useful in problems of classification, learning, and pattern recognition.

Neural networks are widely used in different areas of science. Some of their applications are, the prediction of the rate of penetration in soil drilling operations [44], detection of Parkinson’s disease [45], and wind power generation forecasting [46].

For a more detailed review of ANN theory and its applications, it is recommended to review [47].

## 3. Proposed Methodology

**Figure 4:**
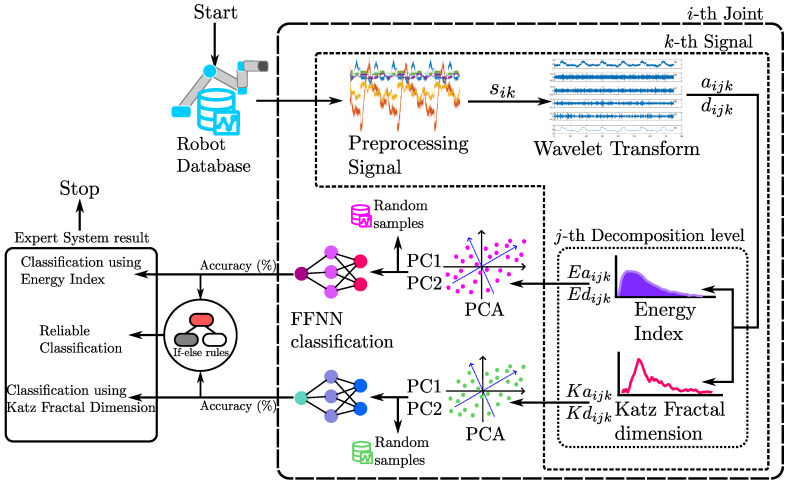
Methodology diagram.

The proposed methodology, to identify the degradation of the robot position through an analysis of the motor’s current, consists of seven stages, presented in Figure 4, and can be summarized as follows: firstly, in the preprocessing stage, for each robot joint *i*, the original signal at different noise levels (5 dB, 10 dB, 15 dB, 20 dB) is used, to give robustness to the method; next, the analyzed signals are decomposed by means of the DWT method, in diverse frequency bands, in order to find patterns or differences associated with the deviation of the robot, since DWT maintains the characteristics of the original signal through the frequency bands. Then, for each signal *k*, and the level of the decomposition *j*, the energy and Katz fractality features are extracted for each approximation ($Ea_{ijk}$ and $Ka_{ijk}$) and detail ($Ed_{ijk}$ and $Kd_{ijk}$) component, with the aim of discovering relevant features in the decomposed signals. Subsequently, the information of each feature is reduced using PCA, also random samples are collected separately for both features in every noise level, for further method validation. The principal components obtained from each feature are used in the training, validation, and testing of an FFNN classifier, for energy and Katz, respectively. Finally, ANNs validation for each noise level’s signals are performed individually, using the random samples collected before. The results of the classifiers based on the Katz and energy indicators are compared, using if-else rules, to provide a double-check diagnostic and obtain a reliable result.

The first stage consists of a robot dataset of current signals for each motor joint. A description of the database is given in the next section.

### 3.1. Database Description

**Figure 5:**
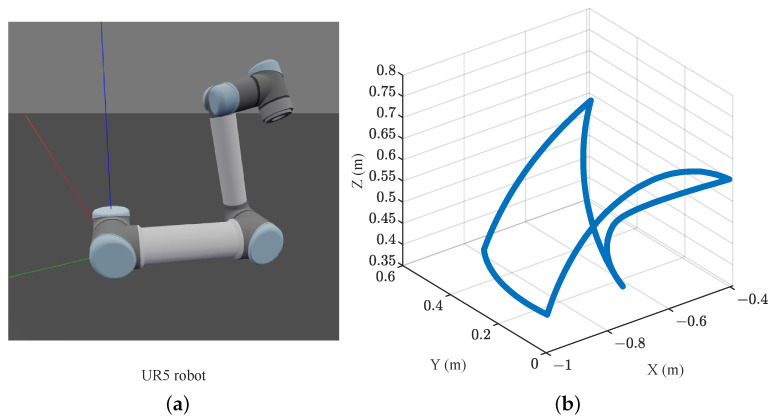
Robot and trajectory. (**a**) UR5 robot, image obtained using the library Robotics Toolbox for Python, available in [48]. (**b**) The trajectory followed by the robot in continuous operation.

For this work, the signals have the following characteristics:

- The robot used to obtain the dataset is a UR5, shown in Figure 5a.
- The current signals from the robot actuators were obtained at the controller level, with a sampling frequency of 125 Hz.
- The signals are captured when the robot performs a continuously repeating trajectory, shown in Figure 5b, with a 4.5 lb mass at the end-effector.
- The first dataset of signals contains three signals when the robot starts cold at 50% of the maximum speed and performs the trajectory continuously.
- The second dataset contains three signals from when the robot has been in continuous operation, performing the same trajectory for approximately 2 h.

In this way, a database with “cold-start” (CS) and “hot-operation” (HO) signals is obtained.

For this work, there is a database for 50% of the speed of the robot and another for 100% of the speed (“half-speed” and “full-speed”, respectively). The database used for this work was published by the National Institute of Standards and Technology (NIST) and is publicly accessible [26].

### 3.2. Experimental Setup

With the information from the database ready, it is proposed to continue with the method as follows:

The signals from the database go through a preprocessing stage, where different noise levels are added, to give robustness to the proposed methodology. The noise levels considered are 5 dB, 10 dB, 15 dB, and 20 dB, and 50 signals are generated for each noise level. This process is repeated for the three signals contained in each dataset, for a total of 150 signals for each condition.

Then, each signal $s_{ik}$, where *i* represents the articulation number, and *k* is the signal number, is processed with the discrete wavelet transform, to separate the signal into different frequency bands. For approximations and details, denoted as $a_{ijk}$ and $d_{ijk}$, respectively, *j* indicates the level of DWT decomposition using discrete Meyer as the wavelet mother.

**Figure 6:**
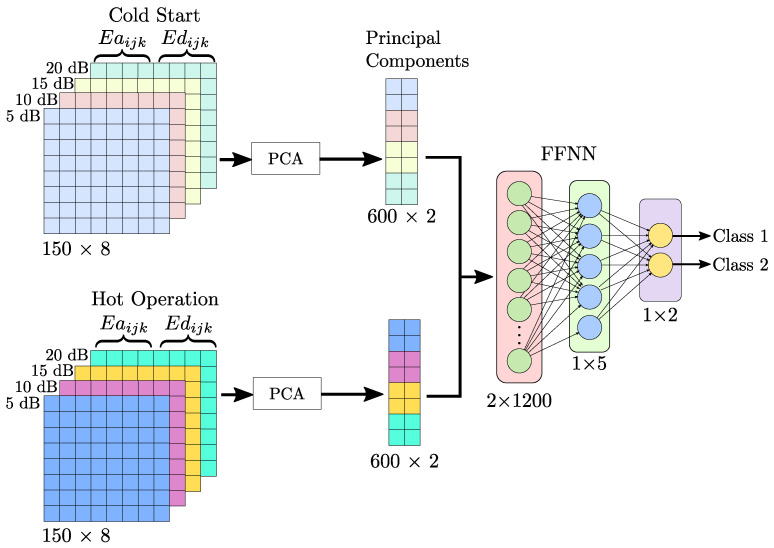
Dataset dimensional reduction and rearrangement for FFNN classification.

Subsequently, for each level of approximation and detail of each signal $s_{ik}$, the energy index ($Ea_{ijk}$ and $Ed_{ijk}$) and Katz fractality ($Ka_{ijk}$ and $Kd_{ijk}$) features are calculated. Due to the DWT separating the original signal in different frequency bands depending on the decomposition level, the number of features increases with a higher level, e.g., for level four, four approximations and four details are obtained for a total of eight characteristics, to calculate for each index. For this reason, and in order to keep the neural network as simple as possible, when the approximations and details of each index for the k=150 signals are calculated, they are arranged in a matrix array for each feature, to reduce the dimension using the PCA algorithm, as shown in Figure 6 for the energy index, the same process is applied to the Katz fractal dimension. In Figure 6, the matrices of features for every noise level are represented with different colors, then, after the PCA reduction, a new matrix with a lower dimension is obtained for each noise level and is represented with the respective color.

In this work, *n* main components, $PC_{n}$. are considered, such that the representability of the original dataset is greater than 50%. This is achieved by using two principal components. At this stage, for each feature, a percentage of the samples for each noise level is randomly selected for further validation of the FFNN. Then, for each index, the principal components’ matrices of all noise levels are arranged in a matrix that is used as input for their respective FFNN, to classify between “CS” and “HO” conditions. In this way, two FFNN classifiers are obtained, and each one uses a different feature.

The neural network configuration consists of an input layer of two neurons. A hidden layer with five neurons and a log-sigmoid activation function is used. Two outputs are contemplated in the neural network (linear activation function), which indicates to which class (“CS” or “HO”) the processed signal belongs. The selection of the samples for the training, validation, and test stages of the neural network is carried out randomly. Then the dataset is divided as follows: for the training stage, 70% of the samples are used, and for the testing and validation stages, 15% are used for each. A summary of the neural network configuration is shown in Table 1 and Figure 6.

Finally, the results obtained with the FFNN classification are stored for later comparison. The results for both classifiers are combined using a simple if-else statement, in order to build an expert system, to obtain a more reliable result. Expert systems are systems that imitate the decision-making process of a human expert [49]. The proposed conditions for the expert system are: if both FFNNs give different results classifying the same signal, the analysis is in an uncertain condition, and it is recommended to repeat the analysis using more signals. Else, both FFNNs give the same result, proceeding to robot examination when the deviation is detected in a joint.

The computer equipment used to implement the methodology has an eight-core ARM64 processor, 8 GB of RAM, 256 GB of hard disk, and runs a UNIX-based operating system.

This methodology is equally implemented for the “half-speed” and “full-speed” datasets. The results of both experiments are presented in the next section.

## 4. Results

The results obtained with the methodology proposed for the half- and full-speed cases are introduced below.

**Figure 7:**
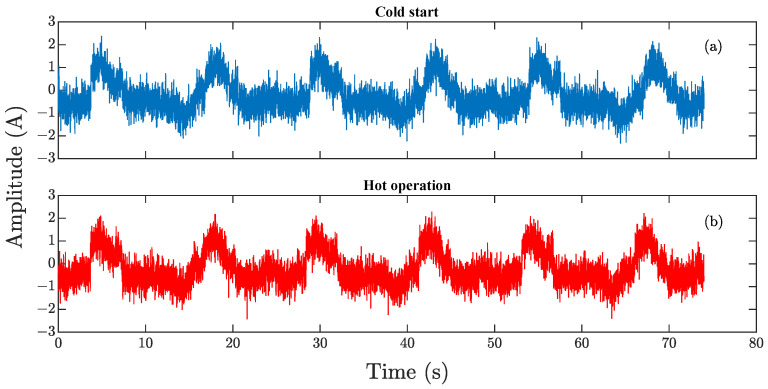
Current signals of joint one with 5 dB noise: (**a**) signal for the robot cold-start state; (**b**) signal for the robot hot-operation state.

Figure 7 shows the graphs of the current signals for joint one with a noise level of 5 dB. Figure 7a shows the “CS” signal, and Figure 7b shows the signal after 2 h of continuous robot operation “HO”. Comparing both graphs, no significant differences are found at first glance. For this reason, a time-frequency analysis is performed using DWT, due to the capacity to conserve the signal characteristics of the different frequency bands. The analysis is performed from levels one to six, but the last two levels have not offered significant information about the phenomenon, due to the very narrow frequency bands, also the amplitude of these bands compared to the other levels is minimal, which indicates that most information is found in the first four levels of decomposition. Equations (3) and (4) are used to calculate the ranges of the frequency bands. It is observed that the frequency ranges are low; for approximations, the ranges are 0 to 0.95 Hz for level six, and 0 to 1.95 Hz for level five. In the case of details, the ranges are 0.97 to 1.95 Hz for level six and 1.95 to 3.92 Hz for level five. For these reasons, and due to the 125 Hz sampling frequency of the signals, a decomposition level j=4 is used in this work.

**Figure 8:**
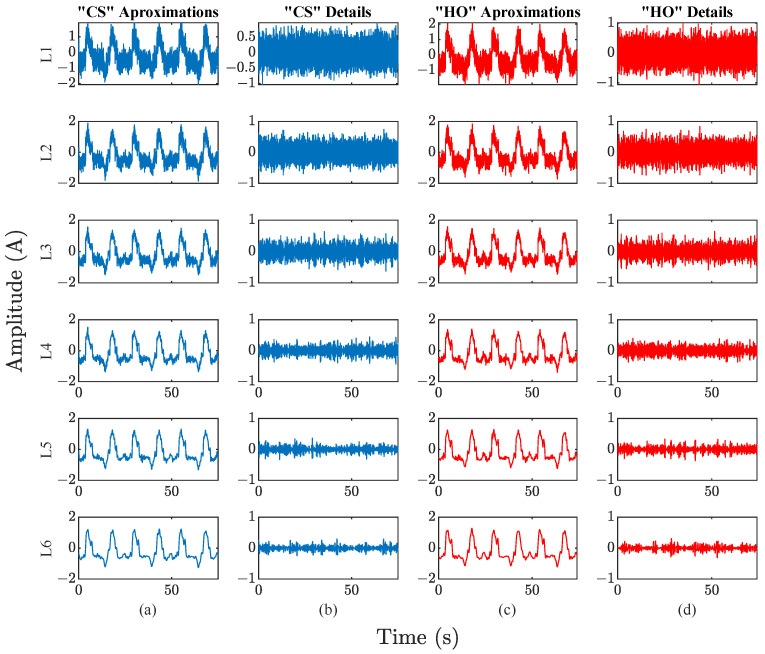
Signals corresponding to the time-frequency analysis of the first joint of the robot, with 5 dB noise, for half-speed case: (**a**,**b**) approximation and details for decomposition level six for the “CS” signal; (**c**,**d**) approximation and details for decomposition level six for the “HO” signal.

Figure 8 shows the results of applying DWT level four to the current signals presented in Figure 7. Figure 8a,b shows the four levels of approximation and details for the CS signal, while Figure 8c,d shows the decomposition levels for the HO signal. In this sense, the approximations and details for “CS” and “HO” do not show a visual pattern that can be associated with position degradation phenomena. For this reason, all frequency bands are analyzed by means of different nonlinear indicators in order to find patterns.

The index calculation is performed for each “CS” and “HO” signal. The objective is to identify the joint that produces deviation in the robot end-effector, by analyzing the current of the motors that move the joints. For this, it is desirable to find a difference between the datasets obtained by calculating the Katz and energy indices in each joint, since this represents a current variation that produces the deviation of the final effect of the robot.

**Figure 9:**
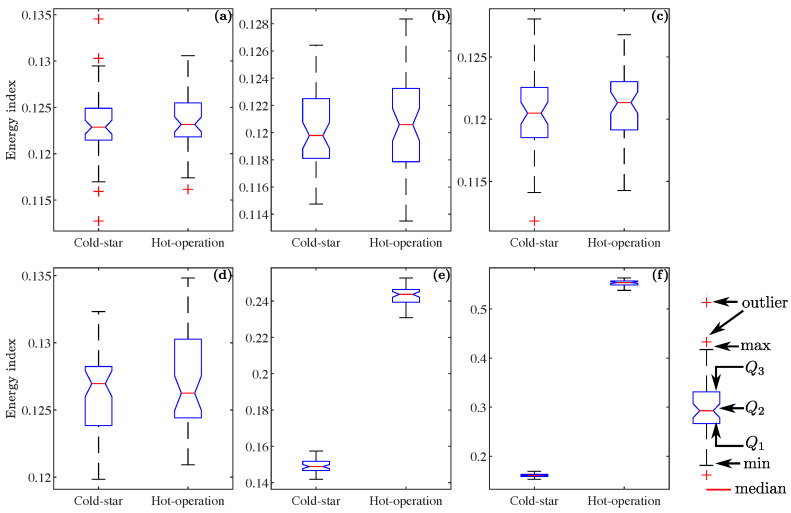
The energy index values from the “cold-start” and “hot-operation” datasets, corresponding to a noise level of 5 dB for level 1 detail: (**a**–**c**) set of values for joints 1 to 3; (**d**–**f**) set of values for joints 4 to 6.

**Figure 10:**
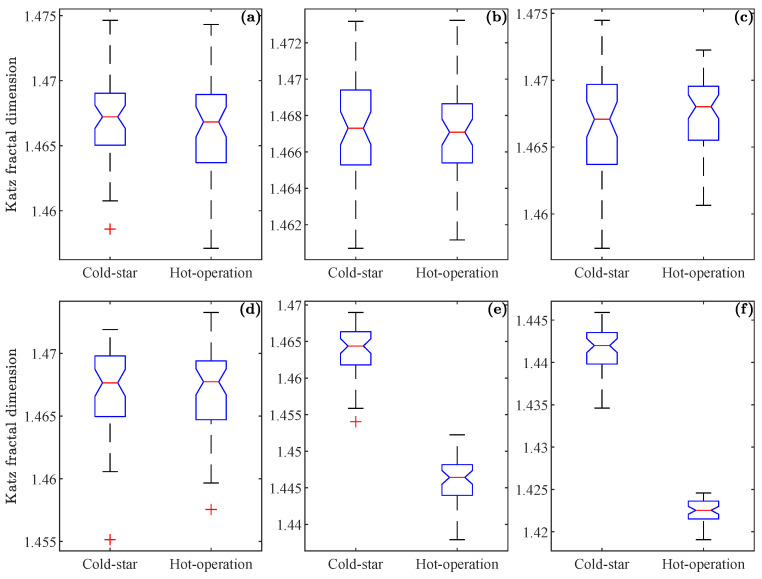
The Katz fractal dimension values from the “cold-start” and “hot-operation” datasets, corresponding to a noise level of 5 dB for level 1 detail: (**a**–**c**) set of values for joints 1 to 3; (**d**–**f**) set of values for joints 4 to 6.

**Figure 11:**
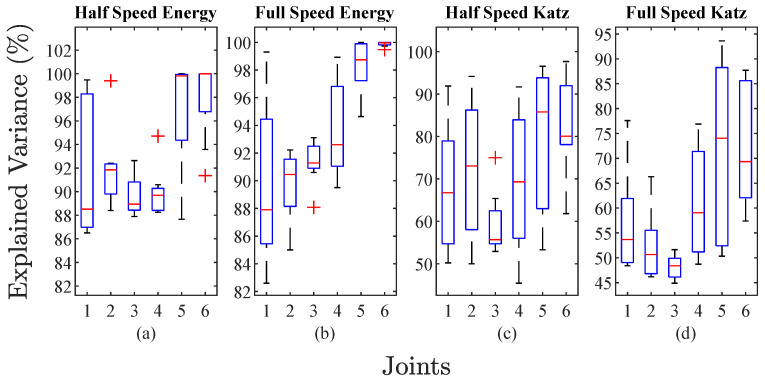
Explained variance accumulated of the two principal components of each joint for half- and full-speed tests: (**a**,**b**) values corresponding to the energy index for all noise levels (5–20 dB); (**c**,**d**) values corresponding to the Katz fractal dimension for all noise levels (5–20 dB).

Figure 9, Figure 10 and Figure 11 use boxplots to show the results; the boxplot presents the dispersion of a group of data using quartile representation. The lower and upper lines in the blue box represent the first and third quartile, respectively (Q1 and Q3), and the red line in the middle of the box stands for the second quartile or median (Q2), the whiskers extend to the minimum and maximum values, and the outlier values (atypical values) are presented as red-crosses. Figure 9 compares the energy indicators of the datasets through “CS” and “HO”; this is for current signals with a noise of 5 dB, from the six joints of the robot. The datasets presented comprise the value of energy in detail one, and are clearly differentiated for joints 5 and 6 (Figure 9e,f). However, the datasets for joints 1 and 4 have a similar spread.

This behavior is also present for the Katz fractality dimension, shown in Figure 10, where the dispersion of the sets for articulations 5 and 6 is differentiated (Figure 10e and Figure 10f, respectively). For joints 1–4, the sets have a similar distance between them. Comparing Figure 9 and Figure 10, it can be observed that the indices proposed, provide a similar result in the extraction of characteristics contained in the signal that cannot be visually detected.

Since only the level one detail information was analyzed, an automatic method for analyzing the remaining levels of details and approximations, carried out using a neural network, was used to classify the current signals of the robot contemplating the four decomposition levels and different noise levels proposed. For this, a data reduction using PCA is implemented.

The representability of the new data is presented in Figure 11; this shows four boxplots, where each one displays values of explained variance for the six robot joints. The explained variance generated by PCA measures the data variation attributive to each principal component. The principal components with higher explained variance are the most significant. Figure 11 presents the sum of the explained variance of the two principal components corresponding to the reduction with PCA of the energy index (Figure 11a,b) and Katz fractal dimension (Figure 11c,d), for the half- and full-speed cases of study, with noise levels from 5 dB to 20 dB. It is observed that the percentage of explained variance for the energy index has values greater than 80%, compared to the percentage of the Katz index, with a minimum value of 45%.

The behavior of the variance dispersion is similar in both cases for the energy index; also, it is observed that joint 3 presents the least dispersion in both cases, and the representativeness of the information is better for the energy index. For the Katz index, the dispersion is higher, in order to obtain a compact range dispersion of the explained variance value, it is necessary to use more principal components; however, to maintain the generality and probe the robustness of the proposed methodology, only two principal components are used, as in the energy index. Subsequently, the new PCA features are used to train and validate an ANN for classification. In Table 1, the configuration of the ANN is presented. The parameters for the ANN are obtained through a trial-and-error calibration process.

**Figure 12:**
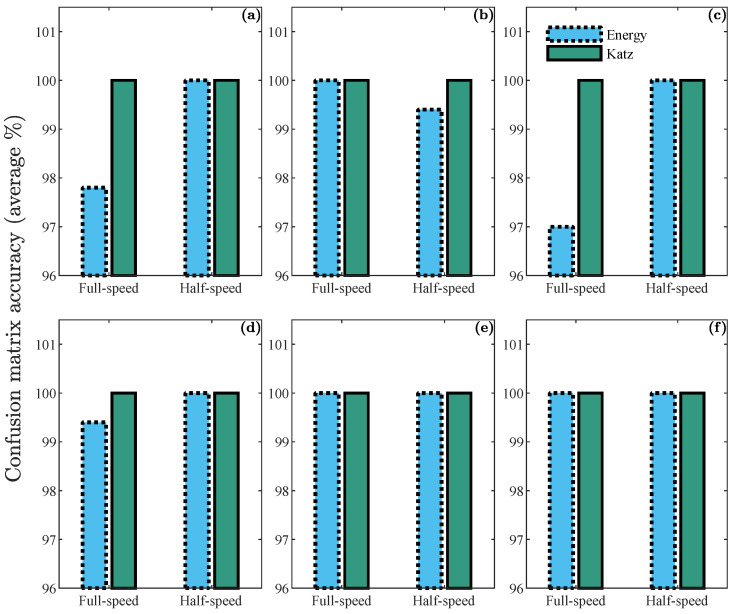
Confusion matrix accuracy of half- and full-speed cases using mixed noise level samples for validation: (**a**–**c**) results corresponding to joints 1 to 3; (**d**–**f**) results corresponding to joints 4 to 6.

**Figure 13:**
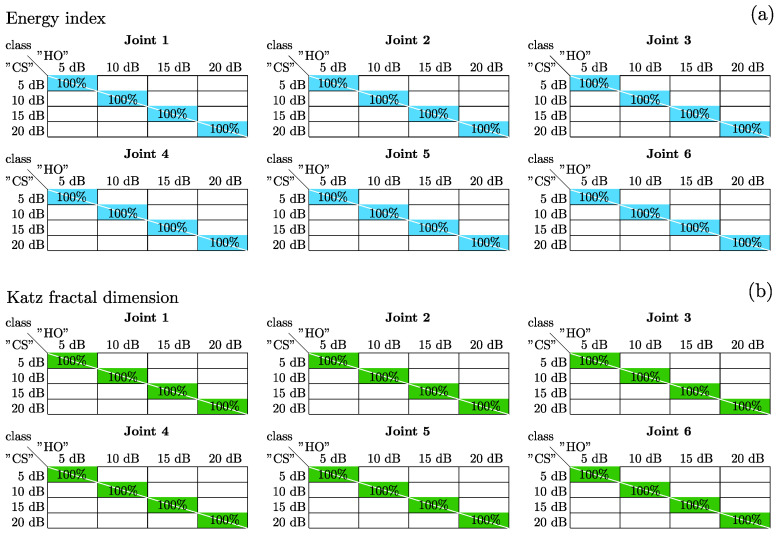
Confusion matrix average accuracy of half- and full-speed cases, using specific noise level samples for validation: (**a**) results corresponding to energy index classification; (**b**) results corresponding to Katz fractality classification.

The results obtained from the classification are shown in Figure 12 and Figure 13. In Figure 12, the average percentage of accuracy of the confusion matrices of the neural networks for features of combined noise levels is presented. Six bar graphs are shown, each corresponding to a robot joint. Each bar graph shows the percentages for the full- and half-speed cases, and every individual bar belongs to an ANN classifier.

The dotted-contour bar shows the percentage corresponding to the neural network that uses the energy index for training. In contrast, the solid-contour bar corresponds to the network that uses the Katz fractality index. In Figure 12e,f, corresponding to joints 5 and 6, it can be seen that the neural network classification has an accuracy of 100%, with both the energy indicator and the Katz fractality indicator. This result is expected when looking at the spread of the dataset in Figure 9 and Figure 10.

Regarding joints 1 to 4 (Figure 12a–d), it is observed that in the case of full-speed with the energy index, the classification obtains results greater than 97%. In contrast, with the Katz fractality index, 100% accuracy is obtained. Finally, in the case of half-speed, an accuracy greater than 99% is obtained for both indicators.

In Figure 13, the average accuracy of the confusion matrix, for validation with specific noise level signals, is shown for the half- and full-speed cases. The results for the energy index are shown in Figure 13a, whereas the results for the Katz fractal dimension are shown in Figure 13b. Both figures are composed of six tables, one for each robot joint; each table has the average precision of the confusion matrix obtained by validating the classifiers using only the corresponding noise level. The average considers the mean between the half- and full-speed cases. The diagonal line in the table indicates the division of the results for the classes “CS” and “HO”. The results showed an accuracy of 100% in the classification, using only specific noise signals for their respective noise levels, in both speed conditions.

Finally, to obtain a more reliable result, the estimated results of the FFNN classifiers, using Katz and energy features for specific noise level signals, are combined to build an expert system using the previously proposed if-else conditions. Due to the obtained results for both classifiers presenting 100% accuracy, the proposed expert system has a 100% accuracy for each joint, in different noise and velocity conditions. In addition, the low time to complete the analysis should be pointed out, as presented in Table 2, the time values correspond to a 25-run evaluation for the full-speed dataset, using the 5 dB noise signals. Table 2 presents the mean time and standard deviation results for each joint, and the proposed method is implemented in the equipment presented previously in the methodology Section 3.2. The average time to perform a joint analysis is 5 s; nevertheless, in addition to this time, it is necessary to add the signal acquisition time of approximately 75 s, to obtain a total of 80 s for each joint analysis. This time value allows the proposed methodology to be considered a robot quick positional health assessment.

## 5. Discussion

The proposed methodology allows the classification of the current signals of the actuators of an industrial robot to determine if there are changes that lead to a deviation in the position of the robot. For this, an expert system is implemented, using the result of both FFNN classifiers.

Among the advantages of the proposed method, an external measuring device is not required to carry out the analysis, since the method is based on the signals obtained at the controller-data level. Besides, if the current signals are acquired with an external data acquisition system, the methodology contemplates the possibility of noise in the signals. On the other hand, when using the DWT, it is possible to select a frequency band to conduct the analysis and reduce the data size and processing time. The proposed indices are highly effective and straightforward to implement. The FFNN architecture used has only five neurons in the hidden layer, which makes it a simple and fast neural network. In addition, combining the results of the FFNN classifiers in an expert system provides a more reliable and robust method for detecting anomalies in the robot joints’ behavior. The results for joint five, where a 100% classification accuracy is obtained, are supported by those reported in [24]. A deviation in this robot joint is shown through a review of the position and velocity graphs. These deviations can produce position jitter in the robot TCP and cause problems in high-accuracy manufacturing tasks.

However, the proposed method is a quick health assessment, so we only obtain information on which joint, in particular, has significant differences in the current signals through nonlinear features. The difference between the initial state concerning the actual state, can present a joint deviation that causes a loss of accuracy in the TCP of the robot. The proposed method does not cover the magnitude of the deviation or its origin (e.g., mechanical or electrical failures). It should also be noted, that the results obtained are preliminary, as they were tested on a database with few signals, and it is important in the future to test on a database with a larger number of signals.

On the other hand, the industrial robot databases with public access are limited, and attend to other problems in the robotics area (generally collisions). In addition, the requirement that the robot movement has to be the same over long time periods is a limitation for database selection. For example, the method reported in [21], where the same dataset used in this work is used, addresses the residual error of the robot’s TCP and does not consider the individual analysis of the joints; therefore, only a qualitative comparison can be made. In this sense, the proposed method can identify in which part of the robot there is a deviation that directly affects the precision of the TCP due to the accumulation of the error through the kinematic chain. Besides, in [24], where the dataset was originally presented, a comparison is made between the position error and the velocity of the robot joint, to determine if there is a deviation. However, no method or model is presented to perform the analysis, although quantitative results are obtained about the deviation and in which joints it is found. Compared to our proposal, its analysis is performed manually and depends on the interpretation and experience of the user to give a result, whereas ours does not provide the magnitude of the deviation, but it is an automated method and can be implemented in different robots, besides, it is not necessary to have a deep knowledge of robotics to obtain a result, because the system delivers a result through a double-checking process. Due to the limited access to robot databases, the objective of this work is the methodological proposal and the demonstration of its reliability, using a publicly accessible robot database.

## 6. Conclusions

Robotic systems are essential elements in modern manufacturing, since they allow for performing diverse tasks, e.g., pick-and-place, welding, machining, and painting, etc. The robot’s accuracy in positioning and orienting itself is essential for precision and quality; however, its accuracy in the process can be degraded by various factors, such as environmental conditions and the wearing of mechanical and electrical components. In this regard, a rapid robotic positional health assessment methodology, to determine if there is position degradation in the joints of an industrial robot after two hours of continuous operation, is introduced in this work. For this purpose, a public database of a UR5 robot is used, where its measured signals are also contaminated with different noise levels, in order to give more robustness to the proposed method. Hence, the current signals of the joints are decomposed in different frequency bands, using DWT. Then, each frequency band approximation and detail, are processed by two nonlinear indicators: energy index and Katz fractality, in order to identify reliable patterns in the analyzed signals. The obtained features are reduced by means of the PCA method and are used to train and validate a set of neural network-based classifiers (one for each feature, energy and fractality). Finally, the outputs of both classifiers are used to form an expert system and deliver a more reliable result.

The obtained results show that the proposed method can classify the robot’s current signals with high accuracy, for the energy and Katz indices, for the two-speed level cases of the robot. For joints five and six, the classification accuracy is 100% for the two-speed cases, with both indicators using samples of all noise levels signals combined. For all analyzed levels of noise, both classifiers have 100% accuracy. Due to the individual results of the classifiers, the combination of the results in the expert system also provides a 100% accuracy, resulting in a more reliable method to detect abnormal behavior in the robot, due to the double-checking process. In addition, the proposed method is helpful in quickly identifying deviating joints (80 s approximately), especially in a serial robot arm, where the error accumulates along the kinematic chain. In contrast with other works that share the use of the same database, such as [21], where the residual error of the TCP is addressed, or [24], where a handmade comparison is made between the position and velocity of the joints to detect deviation; the proposed method can identify, in an automated way, in which part of the robot is the deviation, it does not require modeling of the robot or external sensors to perform measurements, and the method is applicable to robots of different degrees of freedom. The early identification of the deviating joint allows the implementation of robot calibration methodologies and the generation of diagnostic strategies for preventive maintenance of the robot, reducing maintenance times and costs, both valuable resources in the industrial field.

It should be noted that the obtained results should be considered preliminary, since the proposal is evaluated by using a reduced number of tests from the database. For this reason, in future work, it is important to validate the proposed methodology with a larger database, which also includes information about different failures that affect the positional accuracy of the robot. Moreover, it is essential to explore other machine-learning and deep-learning techniques and their application in the generation of fast robot positional health assessment methodologies.

## Figures and Tables

**Table 1:** FFNN configuration parameters.

Parameter	Value
Input layer	2
Neurons in hidden layer	5
Transfer function	log-sigmoid
Output layer	2
Data division	random
Train ratio	70%
Validation ratio	15%
Test ratio	15%
Training	Levenberg–Marquardt
Epoch	5000

**Table 2:** The time information of the 25-run simulation of the proposed method for full-speed signals with 5 dB.

Joints	$q_{1}$	$q_{2}$	$q_{3}$	$q_{4}$	$q_{5}$	$q_{6}$
mean (s)	5.3541	5.4378	5.2729	5.2802	5.3078	5.2812
std (s)	0.2604	0.4894	0.0148	0.0189	0.1020	0.0182

## Data Availability

Not applicable.
